# A poly-L-lysine-functionalized electrochemical immunosensor enables highly sensitive detection of tumor necrosis factor receptor-associated protein 1

**DOI:** 10.55730/1300-0527.3909

**Published:** 2026-06-10

**Authors:** Berfin VURAL, Mustafa Kemal SEZGİNTÜRK

**Affiliations:** Çanakkale Onsekiz Mart University, Faculty of Engineering, Bioengineering Department, Çanakkale, Turkiye

**Keywords:** TRAP-1, biomarker, cancer diagnostics, ITO, PET electrode, electrochemical biosensors

## Abstract

Tumor necrosis factor receptor-associated protein 1 (TRAP-1), a member of the mitochondrial heat shock protein family (HSP90), is essential for protein homeostasis, stress response, and cell cycle. TRAP-1 not only optimizes cellular energy production but also prevents cell death, thereby supporting the survival and proliferation of tumor cells. In addition to cancer, TRAP-1 has been associated with Parkinson’s disease, certain autoimmune disorders, and abnormalities in the kidney and urinary tract. In this study, a unique and useful indium tin oxide-polyethylene terephthalate (ITO–PET)-based electrochemical biosensor is described for detecting the biomarker TRAP-1. The ITO–PET electrode, selected as the working electrode, is a highly advantageous semiconductor electrode material due to its ease of functionalization, low cost, flexibility, and stability. It also stands out as a favorable working electrode due to its ability to detect analytes at very low concentrations. Electrochemical impedance spectroscopy and cyclic voltammetry were used to evaluate the immunosensor, optimize operating parameters, and characterize the biosensing system. In addition to these characterization techniques, single frequency impedance measurements were used to investigate the interactions of anti-TRAP-1 with TRAP-1. To evaluate the clinical applicability of the immunosensor designed, commercially available real human serum samples were analyzed. In conclusion, the biosensor was characterized and shown to be sensitive, reliable, and reproducible across a large detection range (0.004–20 pg/mL).

## 1. Introduction

Tumor necrosis factor receptor-associated protein 1 (TRAP-1), a mitochondrial protein implicated in cancer progression, is gaining attention as a promising biomarker for early diagnosis. As cancer continues to be a leading global health concern, identifying such biomarkers is critical for enabling timely detection and improving clinical outcomes. While various cancer types share the common feature of uncontrolled cellular growth, the underlying triggers may differ significantly [1]. In healthy individuals, regularly dividing cells may undergo disruption due to genetic and epigenetic (environmental) factors, eventually leading to tumor formation [2]. The etiology of cancer is multifactorial, involving genetic predisposition, infections, environmental exposure, and lifestyle factors [3–5]. Cancer is currently the world’s second greatest cause of mortality, trailing only cardiovascular disorders [6]. According to the World Health Organization (WHO)^1^, one in every five persons may develop cancer during their lifetime, with the most commonly diagnosed type being breast cancer in women and lung cancer in men. Furthermore, an estimated 20 million new cancer cases and 9.7 million cancer-related deaths occur each year, highlighting the crucial need for early detection and prevention. Early identification not only improves individual prognoses but also reduces the cost load on healthcare systems [7]. Early identification and intervention can enhance therapeutic outcomes and reduce mortality rates [8].

TRAP-1 is a mitochondrial heat shock protein that plays a role in protein homeostasis, cellular stress response, and cell cycle [9,10]. It assists in protein folding and regulates protein stability and activity [11,12]. According to recent studies, TRAP-1 is overexpressed in a variety of cancers and plays a significant role in altering cancer cell metabolism [13–15]. Its overexpression is linked to improved oxidative stress tolerance and survival pathways in cancer cells [16,17]. TRAP-1 regulates mitochondrial oxidative stress, stabilizes antiapoptotic proteins, and modulates the electron transport chain [18]. Consequently, its elevated expression supports ATP production while inhibiting mitochondria-mediated apoptosis, thereby promoting tumor development [19]. Overexpression of TRAP-1 has been discovered in a number of cancers, including colorectal, gastric, pancreatic, prostate, and glioblastoma [9,20–23]. Beyond cancer, TRAP-1 has also been associated with Parkinson’s disease, certain autoimmune disorders, and abnormalities in the renal and urinary systems [24,25].

Therefore, developing biosensing platforms capable of rapid, sensitive, and reliable detection of TRAP-1 using ITO–PET electrodes is important. Such platforms may make a significant contribution to the early detection of cancer, Parkinson’s disease, and other illnesses. Biomarkers are quantifiable biological molecules that represent biochemical processes occurring in living systems [26]. These molecules—often proteins, nucleic acids, hormones, or small molecules—represent alterations in gene expression, metabolism, enzymatic activity, or protein synthesis [27,28]. A biomarker has a potential to indicate biochemical changes at the cellular level caused by pathological conditions such as cancer, infection, or inflammation [28]. Hence, biomarkers play a significant role in early disease detection [29]. The ability to detect disease-specific biomarkers at early stages using minimally invasive techniques offers prognostic and therapeutic advantages [30,31]. In the present work, TRAP-1 was examined as an intriguing possibility for the early identification of certain cancer types.

Biosensing systems are devices that utilize biological recognition components and physical transducers for recognizing specific analytes associated with health and sickness. Electrochemical biosensors are widely used due to their ease of use, fast response time, high sensitivity, low cost, and potential for miniaturization [32,33]. These features make them suitable for applications in environmental monitoring, biomedical diagnostics, pharmaceutical research, and food safety. Especially in immunoassays, electrochemical biosensors offer sensitive and specific alternatives to traditional methods [34,35]. Electrochemical immunosensors detect antigen–antibody interactions using techniques like amperometry, potentiometry, voltammetry, impedance spectroscopy, and conductometry [36,37]. Conventional immunoanalytical techniques, like the enzyme-linked immunosorbent assay (ELISA) and radioimmunoassay (RIA), are constrained by lengthy processing times, complicated processes, and high costs [38]. In contrast, electrochemical biosensing systems overcome many of these limitations and offer a promising alternative for biomarker detection. ITO–PET electrodes served as the working electrode in the present study. They are conductive, flexible, and transparent materials composed of a PET film coated with indium tin oxide [39]. These electrodes are widely utilized in electrochemical biosensors due to their optical transparency and mechanical flexibility, facilitating integration into wearable and optical systems [40]. They also offer advantages such as low cost, light weight, and easy surface modification. Additionally, compared to conventional rigid electrode substrates, ITO–PET also offers practical advantages in terms of portability, ease of handling, and potential integration into point-of-care sensing platforms.

In the present study, the conception and development of an electrochemical biosensing system is described for the specific and sensitive detection of TRAP-1. It was also aimed to assess the created biosensor’s diagnostic capabilities and contribute to future cancer biomarker detection applications. A novel electrochemical biosensing system was developed using an innovative immobilization technique for TRAP-1 detection. ITO–PET electrodes were chosen as the working electrode because of their optical transparency, high electrical conductivity, and flexibility. The functionality of the biosensing platform designed was assessed using electrochemical impedance spectroscopy (EIS) and cyclic voltammetry (CV).

## 2. Materials and methods

### 2.1. Chemicals and equipment

All chemicals used in the study, including the ITO–PET electrodes employed as working electrodes, were purchased from Sigma-Aldrich (USA), unless otherwise specified. ITO–PET electrodes were cut to dimensions of 5 mm × 20 mm before use. In the three-electrode configuration, a platinum wire served as the counter electrode, and a saturated Ag/AgCl electrode (in 3 M KCl) functioned as the reference electrode. Both auxiliary electrodes were obtained from iBAS (Warwickshire, UK). Electrochemical measurements were performed on a Gamry Reference 600 Potentiostat/Galvanostat (Gamry Instruments, Warminster, PA, USA) linked to a computer running the software Echem Analyst. Measurements were obtained in a redox solution with 5 mM potassium ferricyanide/potassium ferrocyanide (K_3_[Fe(CN)_6_]/K_4_[Fe(CN)_6_], 1:1) produced in 0.1 M KCl. TRAP-1 antigen, anti-TRAP-1 antibody, and 0.5% (w/v) bovine serum albumin (BSA) solutions were made in phosphate buffer solution (PBS) at pH 7.0. The L-lysine solution for electropolymerization was produced in phosphate buffer at pH 8.0. Ultrapure water (18.2 MΩ·cm) was obtained using an Elga PURELAB flex water purification system. All reagents were weighed using a Shimadzu ATX224 analytical balance and pH values were measured using a Fisher Scientific pH meter.

### 2.2. Immobilization procedure for TRAP-1 detection

To remove any potential surface impurities, the ITO–PET electrodes were subjected to a 10-min sequential ultrasonic cleaning operation with acetone, soap solution, and ultrapure water. Following this pretreatment, the electrodes were modified with poly-L-lysine via electropolymerization (Figure S). The electropolymerization process was performed by using a 0.5 mM L-lysine solution prepared in phosphate buffer (pH 8.0). CV was applied in the −1.2 V to +2.0 V range at a scanning rate of 100 mV/s for 10 consecutive cycles.

Following polymerization, each electrode was carefully washed with ultrapure water. To facilitate antibody immobilization, the poly-L-lysine-modified electrodes were incubated in a 1% glutaraldehyde solution for 15 min. Poly-L-lysine, a positively charged polymer bearing multiple −NH_2_ groups, reacts with the aldehyde (−CHO) groups of glutaraldehyde, forming imine (Schiff base) linkages (−C=N−). Subsequently, anti-TRAP-1 antibodies (2.5 ng/mL) were covalently immobilized onto the glutaraldehyde-activated surfaces via similar −NH_2_/−CHO reactions. This step established a stable cross-linked structure between the poly-L-lysine film and the antibody. To block unreacted aldehyde sites and minimize nonspecific adsorption, the electrodes were immersed in 0.5% (w/v) (BSA) for 1 h. This blocking step was essential to ensure selective antigen–antibody recognition. The resulting ITO–PET-based immunosensors were stored at 4°C. The Scheme provides a schematic illustration of the immobilization method.

### 2.3. Electrochemical measurements

EIS and CV were employed throughout the immobilization, optimization, and characterization studies of the ITO–PET-based biosensor. The poly-L-lysine electropolymerization was performed using CV, guided by preliminary optimization trials and literature precedent. The electropolymerization was carried out in a phosphate buffer solution (pH 8.0) with 0.5 mM L-lysine. CV parameters included a potential range of −1.2 V to +2.0 V, a scan rate of 100 mV/s, and a total of 10 cycles.

In the EIS measurements, the DC potential applied was 0 V and the AC amplitude was 10 mV. Impedance measurements were taken at frequencies ranging from 50,000 to 0.05 Hz. CV measurements were performed with a potential range of −0.5 V to +1 V, a 10 mV step size, and a scan rate of 100 mV/s. The EIS and CV investigations were carried out using a redox probe solution comprising 5 mM ferri/ferrocyanide solution. The main parameter assessed in the EIS measurements was the charge transfer resistance (Rct), which was derived from the semicircular area of the Nyquist plots utilizing the equivalent circuit model. Rct is one of the most important electrochemical parameters, because it directly reflects the interfacial electron transfer behavior at the electrode surface. During the stepwise fabrication of a biosensor the surface modifications and biomolecular interactions on the surface of the electrode lead to changes in electron transfer kinetics reflected as a change in Rct values. Specifically, the binding of the antibody–antigen results in an additional insulating biolayer on the electrode surface, resulting in an increase in charge transfer resistance. Therefore, the monitored changes in the Rct allowed the sensitive detection of TRAP-1 in the proposed biosensor platform. In the equivalent circuit analysis, in addition to Rct, solution resistance, constant phase element, and Warburg impedance (Zw) were considered to better describe the electrochemical behavior of the system.

#### 2.3.1. Poly-L-lysine functionalization of ITO-PET electrodes via electropolymerization

Electropolymerization is a technique in which a monomer undergoes polymerization upon electrical stimulation [41]. In the present study, L-lysine monomers were electropolymerized onto the surface of ITO–PET electrodes to form a polymeric film of poly-L-lysine. The use of poly-L-lysine in this biosensing system was intended to functionalize the ITO–PET surface and to enhance its effective surface area for TRAP-1 detection. Electropolymerization with poly-L-lysine onto the ITO–PET electrodes serves as a critical strategy for preparing the surface for subsequent biological interactions.

L-lysine monomers had to dissolve in a pH 8.0 PBS before being electropolymerized. During electropolymerization, it is assumed that the amino groups of L-lysine monomers participate in radical polymerization.

The poly-L-lysine film formed on the surface of electrodes imparts a positive surface charge, which facilitates the immobilization of the anti-TRAP-1 biomolecule. Due to its structure containing multiple amino groups, the poly-L-lysine layer provides a suitable platform for covalent attachment and surface modification.

## 3. Results and discussion

### 3.1. Steps for immobilizing anti-TRAP-1 onto the ITO-PET surface

**Figure 1:**
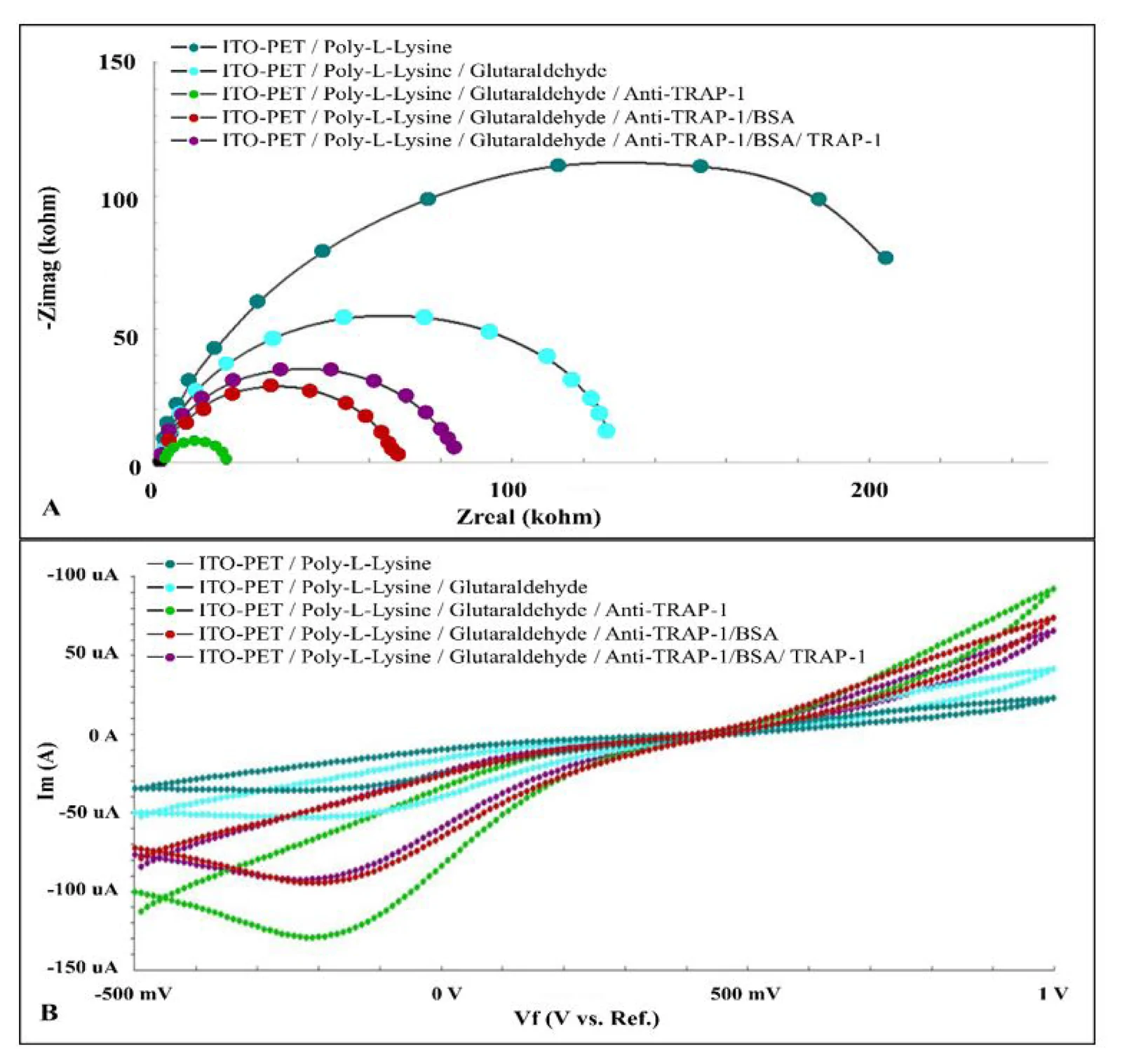
Electrochemical characterization of the immobilization steps of the biosensing system [(A) EIS spectra and (B) CV voltammograms].

Figures 1A and 1B show the EIS and CV data obtained during the immobilization stages of the TRAP-1 biosensor developed. The initial modification of the ITO–PET electrode began with the polymerization of L-lysine. After cleaning, the ITO–PET electrodes were coated with positively charged poly-L-lysine via electropolymerization. This electropolymerization procedure produced positively charged amine groups on the surface of the electrodes. EIS experiments after electropolymerization with poly-L-lysine revealed high charge transfer resistance values. The reason could be related to changes in electron transfer kinetics, or the film thickness. The polymeric film formed on the surface with poly-L-lysine may impede electron transfer, thereby reducing the conductivity of the surface. The increase in electron transfer resistance is observed as an increase in the Nyquist plot during impedance measurements. The formation of a dense poly-L-lysine film could hinder the access of redox couples (Fe^2+^/Fe^3+^) to the electrode surface, slowing down electron transfer. This phenomenon can be interpreted as the formation of a diffusion barrier on the ITO–PET electrode surface, making charge transfer more difficult as a result of electropolymerization.

In the second immobilization step, cross-linking with glutaraldehyde, a decrease in resistance was observed. This can be explained by the rearrangement of the poly-L-lysine layer on the surface due to its reaction with glutaraldehyde, or an increase in its permeability. Furthermore, changes in the coating homogeneity and film thickness may have facilitated the access of redox couples to the surface. A decrease in resistance was also observed during the next stage of immobilization, which involved the anti-TRAP-1 antibody. Contrary to expectations, the decrease in resistance might have been due to the facilitation of the diffusion of the ferri/ferro redox probe due to the interaction with anti-TRAP-1. While it would be expected that this interaction would increase resistance on the surface, it may have actually facilitated charge transfer as a result of surface changes. Specifically, the increase in the gaps on the ITO–PET electrode surface or the facilitation of charge transfer via the anti-TRAP-1 interaction could have led to a decrease in the semicircle diameter of the Nyquist plot.

In the final immobilization step, blocking with BSA, an increase in resistance was observed. BSA protein was used to block the amine groups in the poly-L-lysine structure that could not interact with the antibody, preventing nonspecific binding. As expected, binding of the large molecular BSA protein to the surface resulted in an increase in resistance. This can be attributed to the increased insulating effect on the ITO–PET electrode surface following binding of the BSA protein. Once the immobilization steps of the ITO–PET electrodes were completed, the detection of TRAP-1 protein could be performed. Following the incubation of the ITO–PET electrodes with TRAP-1, an increase in resistance was observed, as expected. This increase can be attributed to antigen-antibody interactions, which complicate charge transfer to the working electrode. The increase in the semicircle diameter of the Nyquist plot in the EIS curves indicates that the target molecule, TRAP-1, successfully bound to the surface, demonstrating that the designed biosensor is capable of detecting the analyte.

To confirm the EIS measurements obtained at each immobilization step, CV measurements were also conducted. In CV measurements, a reduction in anodic and cathodic peak currents is expected when electron transfer on the electrode surface is restricted. When the collected EIS and CV data are reviewed in light of this knowledge, it is discovered that the CV measurements confirm the EIS results. The immobilization process resulted in the development of an ITOPET electrode-based biosensor device that accurately detects the TRAP-1 protein.

### 3.2. Optimization of critical parameters in immunosensor performance

**Figure 2:**
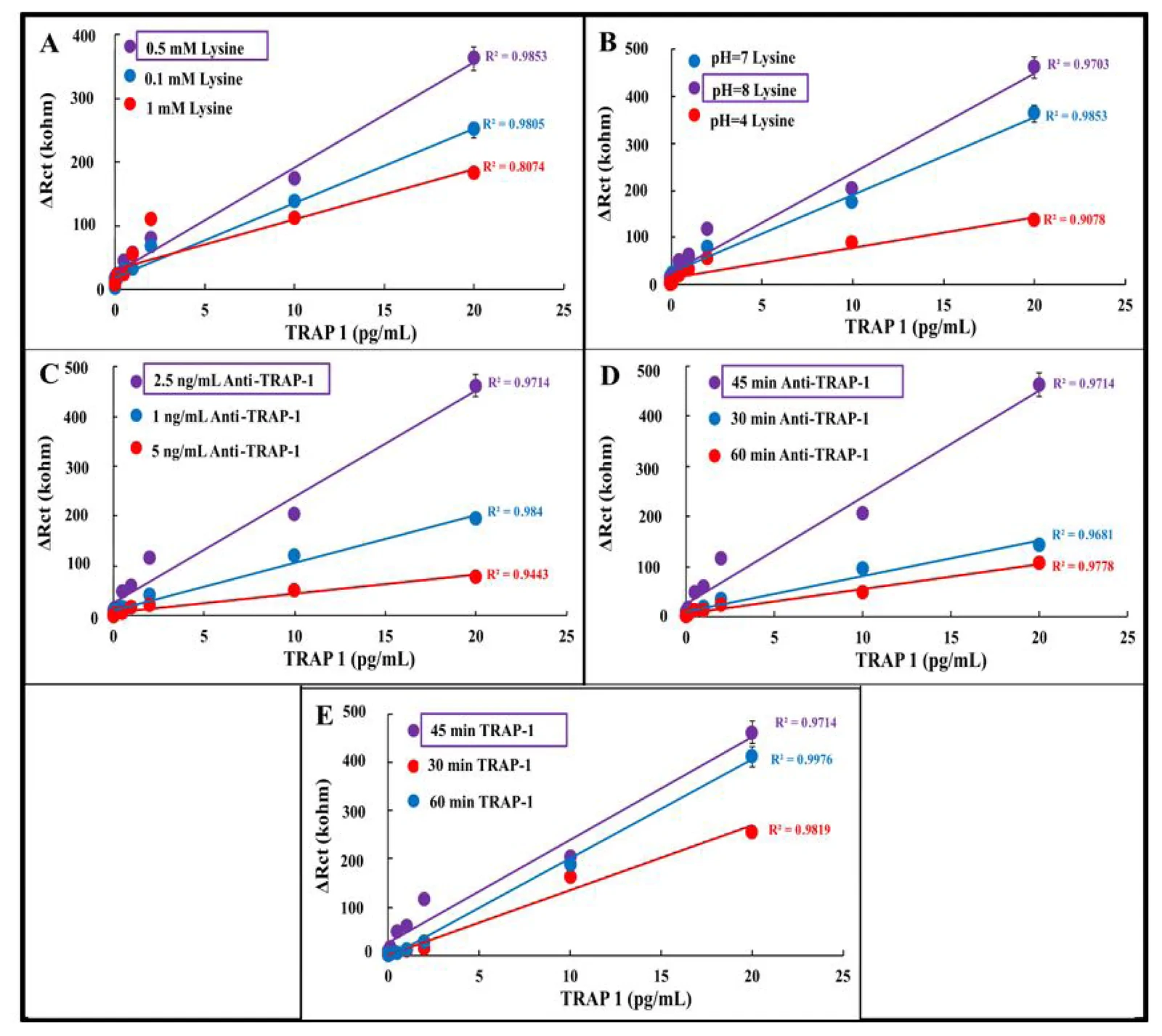
A)The effect of the L-lysine concentration on biosensor response. (●: 0.5 mM L-lysine ●: 0.1 mM L-lysine ●: 1 mM L-lysine). B)The effect of the L-lysine pH value on biosensor response (●: pH 8.0 L-lysine ●: pH 7.0 L-lysine ●: pH 4.0 L-lysine). C)The effect of the anti-TRAP-1 concentration on biosensor response (●: 2.5 pg/mL anti-TRAP-1 ●: 1 pg/mL anti-TRAP-1 ●: 5 pg/mL anti-TRAP-1). D) The effect of the anti-TRAP-1 incubation time on biosensor response (●: 45-min anti-TRAP-1 ●: 30-min anti-TRAP-1 ●: 60-minanti-TRAP-1). E) The effect of the TRAP-1 incubation time on biosensor response (●: 45-min TRAP-1 ●: 30-min TRAP-1 ●: 60-minTRAP-1).

In the development of a biosensor system, determining the optimal conditions for each immobilization step is crucial. To ensure the sensitivity and reproducibility of the – immunosensor developed, several parameters were optimized, including the concentration and pH of the L-lysine solution, the concentration of anti-TRAP-1, and the incubation times for both anti-TRAP-1 and TRAP-1. Poly-L-lysine enables the formation of an appropriate biosensing platform on ITO–PET electrodes for surface modification and antibody immobilization. Therefore, optimizing the L-lysine concentration is a critical step. To optimize the L-lysine concentration for electropolymerization-based coating, three concentrations were tested (0.1 mM, 0.5 mM, and 1 mM), and the corresponding calibration plots were compared (Figure 2A). Among the concentrations tested, the 1 mM L-lysine solution resulted in the lowest sensor response, possibly due to the formation of an overly dense polymer film on the electrode surface that hindered efficient anti-TRAP-1 immobilization and TRAP-1 binding. The 0.1 mM poly-L-lysine solution also led to weak signals, likely indicating insufficient polymer film formation. Based on the comparison of linear responses, 0.5 mM L-lysine was selected as the optimal concentration, as it enabled a homogeneous and functional poly-L-lysine film.

The pH of the L-lysine solution has a considerable impact on the effectiveness of electrode modification. During electropolymerization, optimal ionization of L-lysine monomers is essential for effective adsorption onto the electrode and subsequent film formation. Therefore, L-lysine solutions at pH 4.0, 7.0, and 8.0 were evaluated and compared (Figure 2B). L-lysine, a basic amino acid, contains an α-carboxyl group, an α-amino group, and an additional amino group on the side chain. Their approximate pKa values are listed in Table S. At low pH values (e.g., pH 1.0), all amino groups are protonated, giving the molecule +2 charge. As the pH increases (e.g., pH 2.2), the carboxyl group deprotonates, reducing the charge to +1. At 9.0 pH, the α-amino group loses a proton, and the molecule becomes neutral. Above pH 10.5, the side-chain amino group deprotonates, rendering the molecule negatively charged. The chemical behavior of poly-L-lysine closely resembles that of L-lysine. However, the ionization state of the side-chain amino groups becomes more critical, particularly during film formation. At pH values below 10.5, these side chains are protonated (−NH_3_^+^), maintaining a positive charge. To preserve solubility and charge, only pH values below 10.5 were considered. The phosphate buffer at pH 4.0 likely created an overly acidic environment that hindered polymerization, as supported by the lowest Rct values in the EIS results. At pH 7.0, slightly higher Rct values were observed, possibly due to partial deprotonation of carboxyl groups facilitating polymerization. The best response was achieved with pH 8.0, likely due to increased availability of free amino groups that facilitated film formation. More basic conditions (pH ≥ 9.0) were excluded, as deprotonation of the α-amino group might reduce reactivity and hinder polymerization. Accordingly, pH 8.0 was chosen as the optimal pH for L-lysine polymerization.

The concentration of immobilized antibody on the electrode surface directly affects the biosensor’s sensitivity, selectivity, and efficiency. Therefore, three anti-TRAP-1 concentrations (1 ng/mL, 2.5 ng/mL, and 5 ng/mL) were tested, and the resulting calibration plots were compared (Figure 2C). The biosensor prepared with 5 ng/mL anti-TRAP-1 showed lower sensitivity, possibly due to surface saturation or steric hindrance. The 1 ng/mL anti-TRAP-1 concentration also resulted in a relatively weak response, indicating inadequate antigen–antibody interactions. Based on the highest ΔRct values, 2.5 ng/mL was selected as the optimal anti-TRAP-1 concentration.

Antibody incubation time is another critical factor for achieving stable and specific immobilization. To determine the optimal duration, anti-TRAP-1 was incubated for 30, 45, and 60 min on the electrodes. The resulting linear plots are shown in Figure 2D. ΔRct values were lowest at 30 min, suggesting insufficient time for effective immobilization. At 60 min, a decline in response was observed, possibly due to desorption or nonspecific interactions. The highest ΔRct values were obtained at 45 min, which was therefore selected as the optimal anti-TRAP-1 incubation time.

Finally, the TRAP-1 incubation time was optimized. ITO–PET electrodes were incubated with TRAP-1 for 30, 45, and 60 min. Calibration plots are provided in Figure 2E. Although ΔRct values for 45 and 60 min were similar, the electrodes incubated for 45 min exhibited slightly stronger signals. In contrast, the 30-min incubation resulted in the lowest ΔRct values. Thus, the optimal TRAP-1 incubation time was determined to be 45 min.

### 3.3. Analytical characterization of the TRAP-1 immunosensor

**Figure 3:**
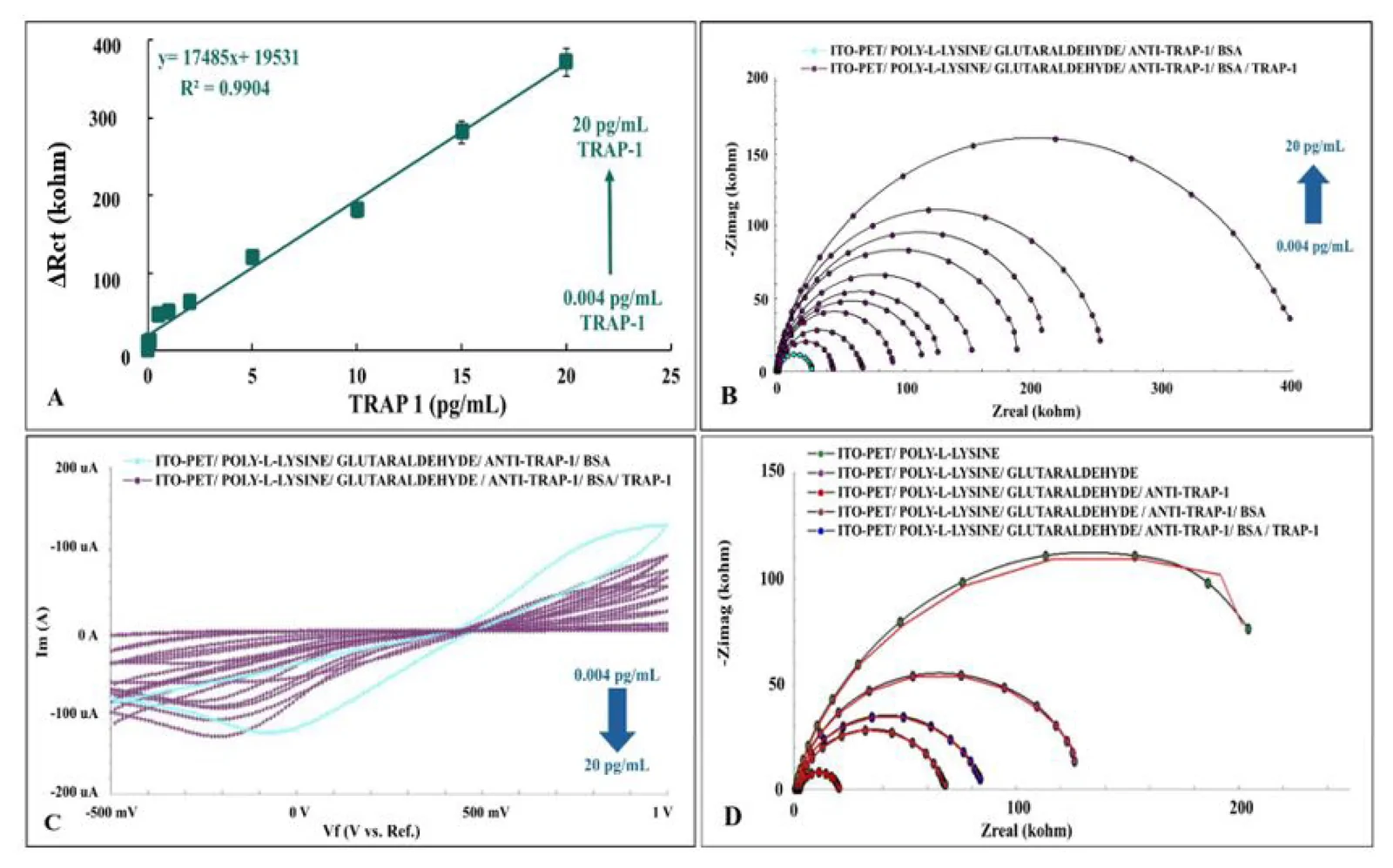
A) Calibration curve of the TRAP-1 immunosensor. B) EIS measurements with increasing TRAP-1 concentrations. C) CV measurements with increasing TRAP-1 concentrations. D) Kramers-Kronig transforms of the proposed immunosensor.

Under optimized conditions, 10 different ITO–PET electrodes were incubated for 45 min with 10 different TRAP-1 concentrations ranging from 0.004 to 20 pg/mL. Antigen–antibody interactions occurring on the electrode surfaces were monitored using EIS and CV. Based on these measurements, a calibration curve for the TRAP-1 biosensor was plotted. The graph is presented in Figure 3A. The resultant data showed a linear detection range of 0.004 to 20 pg/mL. The high R^2^ value obtained from the calibration plot indicates a strong correlation between the biosensor signal and TRAP-1 concentrations. Furthermore, the biosensor was capable of producing consistent and reliable signals across a wide TRAP-1 concentration range. Notably, a distinct signal increase was observed even at very low concentrations such as 0.004 pg/mL, highlighting the sensitivity of the biosensor. The immunosensor showed a linear response over the concentration range of 0.004–20 pg/mL. Since independent blank measurements were not available for a statistically robust LOD/LOQ calculation, the lowest experimentally tested concentration that produced a distinguishable analytical response was reported as the minimum detectable concentration, corresponding to 0.004 pg/mL.

Figures 3B and 3C illustrate the changes in EIS and CV responses with increasing TRAP-1 concentrations. In the Nyquist plots, increases in the Rct values were observed with rising analyte concentrations. Similarly, decreases in anodic and cathodic peak currents of the cyclic voltammograms were observed with increasing TRAP-1 levels. These measurements in both EIS and CV data demonstrate the formation of antibody–antigen interactions on the biosensor surface.

The Kramers-Kronig transformation was used to determine the sensitivity of electrochemical impedance spectra to noise variations generated by external influences during measurement. Using this mathematical method, the reliability of the developed TRAP-1 biosensor is tested. The results are presented in Figure 3D. The close agreement between the experimental data and theoretical predictions confirms the validity of the impedance measurements and the electrochemical reliability of the biosensing system.

Repeatability is a critical metric indicating the stability and consistency of a biosensor system [41]. For determining this, ITO–PET electrodes produced under ideal circumstances were incubated with 10 pg/mL TRAP-1 for 45 min. The electrochemical responses of these electrodes were analyzed to assess repeatability. The average detected concentration was calculated as 10.077 pg/mL, with a coefficient of variation of 4.452% and a standard deviation of ±0.449 pg/mL. The similarity in ΔRct values obtained from 20 independently fabricated electrodes confirms the repeatability and standardization of the fabrication protocol. These findings demonstrate that the biosensor system developed offers high repeatability and operational consistency.

**Figure 4:**
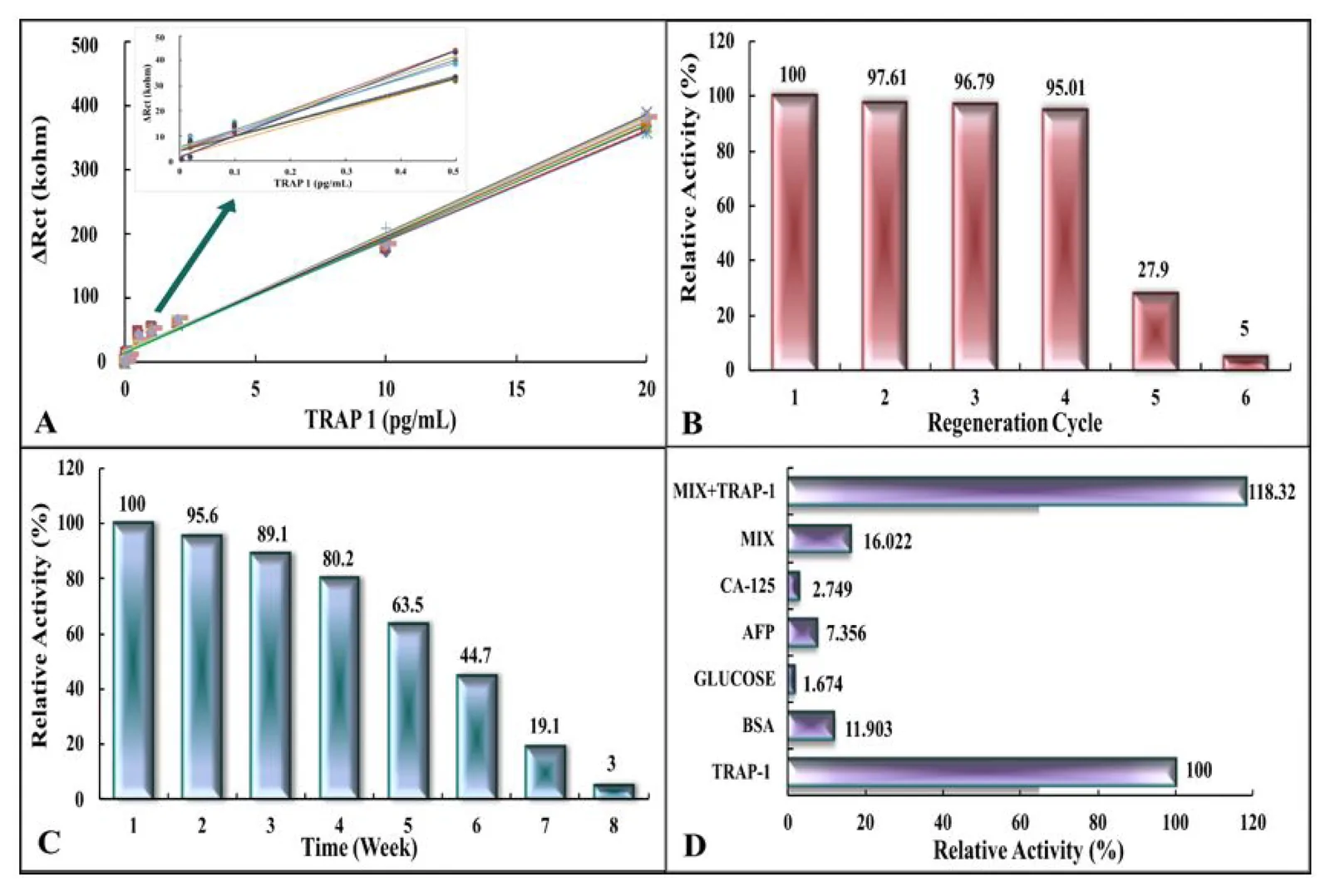
A) Reproducibility study, B) Regeneration experiment, C) Storage stability, D) Selectivity study for the TRAP-1 immunosensor.

A reproducibility study was conducted to assess the consistency and reliability of the immunosensor fabrication process. High reproducibility in a biosensing system indicates consistent performance and analytical reliability. Calibration curves obtained from independently prepared TRAP-1 immunosensors under optimized conditions are presented in Figure 4A. The comparison revealed that the biosensor responses were very constant across all biosensor systems produced, supporting the biosensor’s repeatability.

To assess the reusability of the immunosensor, a regeneration study was performed. An ITO–PET electrode prepared under optimized conditions was first incubated with TRAP-1 antigen and analyzed via EIS. The same electrode was then immersed in a 0.1% hydrochloric acid (HCl) solution for 5 min to disrupt weak interactions such as hydrogen bonding and van der Waals forces between the antibody and antigen. Rct decreased after HCl treatment, nearing the value obtained after the BSA blocking stage. This similarity suggests successful removal of TRAP-1 antigen from the surface without significant loss of electrode integrity. After regeneration, the same electrode was reincubated with 1 pg/mL TRAP-1 for 45 min, followed by another EIS measurement. An increase in Rct confirmed effective rebinding. This regeneration cycle was performed five times. The biosensor maintained stable responses for the first four cycles; however, after the fourth regeneration, physical degradation of the electrode surface was observed. The biosensor could be effectively regenerated up to four times before noticeable performance loss. The regeneration data are illustrated in Figure 4B. These results also demonstrate the durability of the immobilization steps of the proposed biosensor.

Shelf-life research was carried out to determine the biosensor’s stability over time. Biosensor performance was monitored weekly, and the results are presented in Figure 4C. During the first 4 weeks, the biosensor retained stable activity, with signal retention decreasing from 100% to 80.2%. However, a sharp decline in sensor activity was observed after the fifth week. This reduction may result from gradual degradation of the poly-L-lysine film or desorption of biomolecules from the ITO–PET electrode surface due to environmental conditions. The data suggest that the biosensor retains acceptable performance for up to 4 weeks under appropriate storage conditions.

Selectivity was evaluated to assess the biosensor’s specificity for TRAP-1 in the presence of other biological molecules commonly found in human serum. As shown in Figure 4D, the immunosensor exhibited high specificity toward TRAP-1. Interference studies revealed minimal nonspecific adsorption; BSA introduced 11.903% interference, possibly due to its use as a blocking agent and its large protein size. Glucose caused only 1.674% interference, indicating negligible cross-reactivity. Other serum proteins such as AFP and CA125 caused 7.356% and 2.749% interference, respectively, further confirming the biosensor’s selectivity. In the absence of TRAP-1, a mixture of biological components produced 16.022% interference, likely due to synergistic adsorption effects and residual BSA interactions. In contrast, the mixture containing TRAP-1 showed a signal increase of 118.2%, demonstrating the biosensor’s strong and specific recognition ability. These results confirm that the TRAP-1 immunosensor developed is minimally affected by potential interfering species and exhibits highly specific molecular recognition for its target analyte.

### 3.4. Single-frequency impedance (SFI) analysis for real-time monitoring

**Figure 5:**
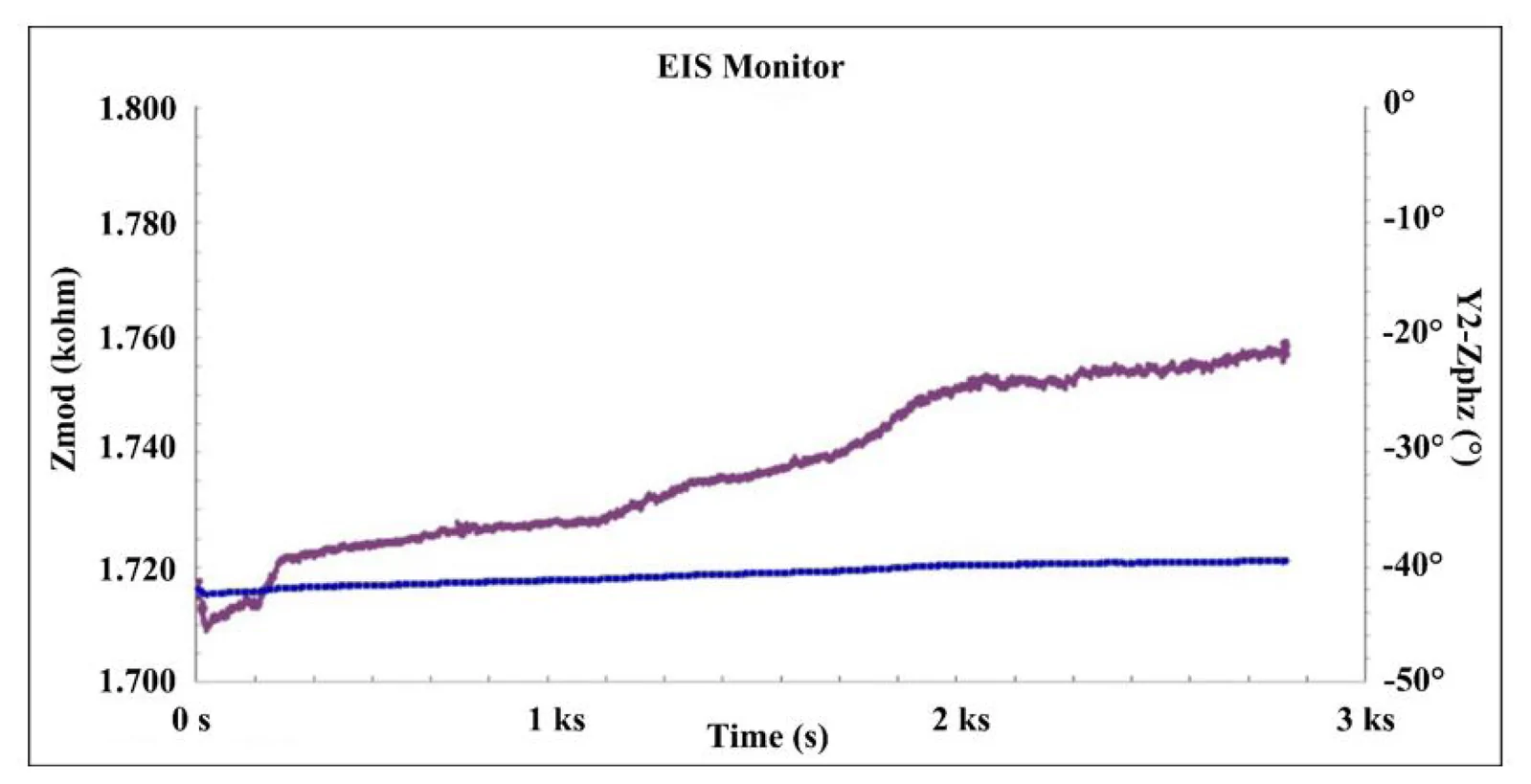
SFI analysis of the TRAP-1 immunosensor ( 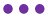: change in impedance, 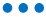: change in phase angle).

EIS measures the biosensor system after biocomponent incubation, while SFI monitors changes on the ITO–PET electrode surface caused by antigen-antibody interactions during the 45-min incubation period. The fixed frequency value for the SFI measurement was selected as 198.6 Hz from the Bode plot. The 45-min SFI measurement was performed in phosphate buffer (pH 7.0). To observe the real-time response of the TRAP-1 biosensing system toward its target molecule, an SFI study was conducted. The SFI measurement diagram of the ITO–PET-based biosensor is presented in Figure 5. Over time, the impedance value recorded at the beginning of the measurement was observed to increase steadily. This rise in impedance indicates the binding of biomolecules to the electrode surface. Additionally, the phase angle remained constant throughout the measurement, suggesting that no significant capacitive interference occurred during the biosensing process. These findings collectively confirm the reliability and stability of the biosensor’s signal in detecting TRAP-1 in real time.

SFI measurements served as an effective electrochemical tool to assess the alterations at the electrode interface during the biosensing process simultaneously. In contrast to full-spectrum EIS analysis, SFI measurements enable the observation of impedance variations at a specific frequency where the biosensor’s response is maximally sensitive to biomolecular interactions. In the present study, we utilized SFI measurements to monitor impedance variations related to TRAP-1 binding events simultaneously.

### 3.5. TRAP-1 analysis using commercial human serum samples

It is important to test the applicability of the immunosensor in clinical studies with commercial human serum samples. Five distinct human serum samples were tested to determine how the proposed TRAP-1 immunosensor responded to commercial human serum samples. The biosensor’s reaction to human serum was evaluated using the standard addition method. For the standard addition method, TRAP-1 antigen was added to commercial human serum samples at concentrations of 0.5 and 2 pg/mL. In order to evaluate these samples in the linear range of the immunosensor developed, they were diluted 1000-fold before standard addition. Within the scope of the present study, parameters such as relative standard deviation and recovery rates were calculated to assess the accuracy and reliability of the biosensor in complex biological media. The corresponding results are presented in the Table. The experimental findings indicated that the biosensor provided accurate measurements with low error margins in serum matrices. These results suggest that the biosensing system developed has strong potential for practical application in clinical diagnostics.

## 4. Conclusion

The detection of TRAP-1, a potential biomarker associated with a wide range of diseases—from various types of cancer to neurodegenerative, renal, and cardiovascular disorders—holds critical diagnostic significance. In the present study, an electrochemical biosensing platform was developed for the detection of TRAP-1 protein and its analytical performance was systematically evaluated. Each immobilization step of the designed immunosensor was optimized through a successful surface modification strategy. The thin poly-L-lysine film formed via electropolymerization was shown to be effectively deposited on the ITO–PET electrode surface, enhancing both the stability and analytical performance of the biosensing system. Electrochemical analyses demonstrated that the immunosensor provided reliable responses even at low TRAP-1 concentrations. The biosensor exhibited high accuracy and repeatability in laboratory-based experiments and enabled cost-effective, sensitive, and rapid detection of the TRAP-1 protein. The newly developed poly-L-lysine-functionalized TRAP-1 immunosensor, compared with the earlier 3-GOPS-based platform [42], introduces an electropolymerized functional interface, has improved analytical sensitivity, enables more comprehensive selectivity studies and mathematically validated impedance analysis, and involves a substantially simplified fabrication strategy. In summary, the biosensor developed in the present study offers a practical fabrication method along with strong signal stability, high sensitivity, excellent repeatability, and the potential for clinical adaptation. These features collectively support its feasibility for future diagnostic applications.

## Supplementary Data

**Table S:** Approximate pKa values of the groups found in the structure of L-lysine.

Chemical groups	pKa value (≈)
α-Carboxyl (−COOH) group	2.2
α-Amine (−NH _2_) group	9.0
Side chain Amine (−NH _2_) group	10.5

Figure S Electropolymerization process of ITO-PET electrodes with L-lysine

## Figures and Tables

**Scheme:**
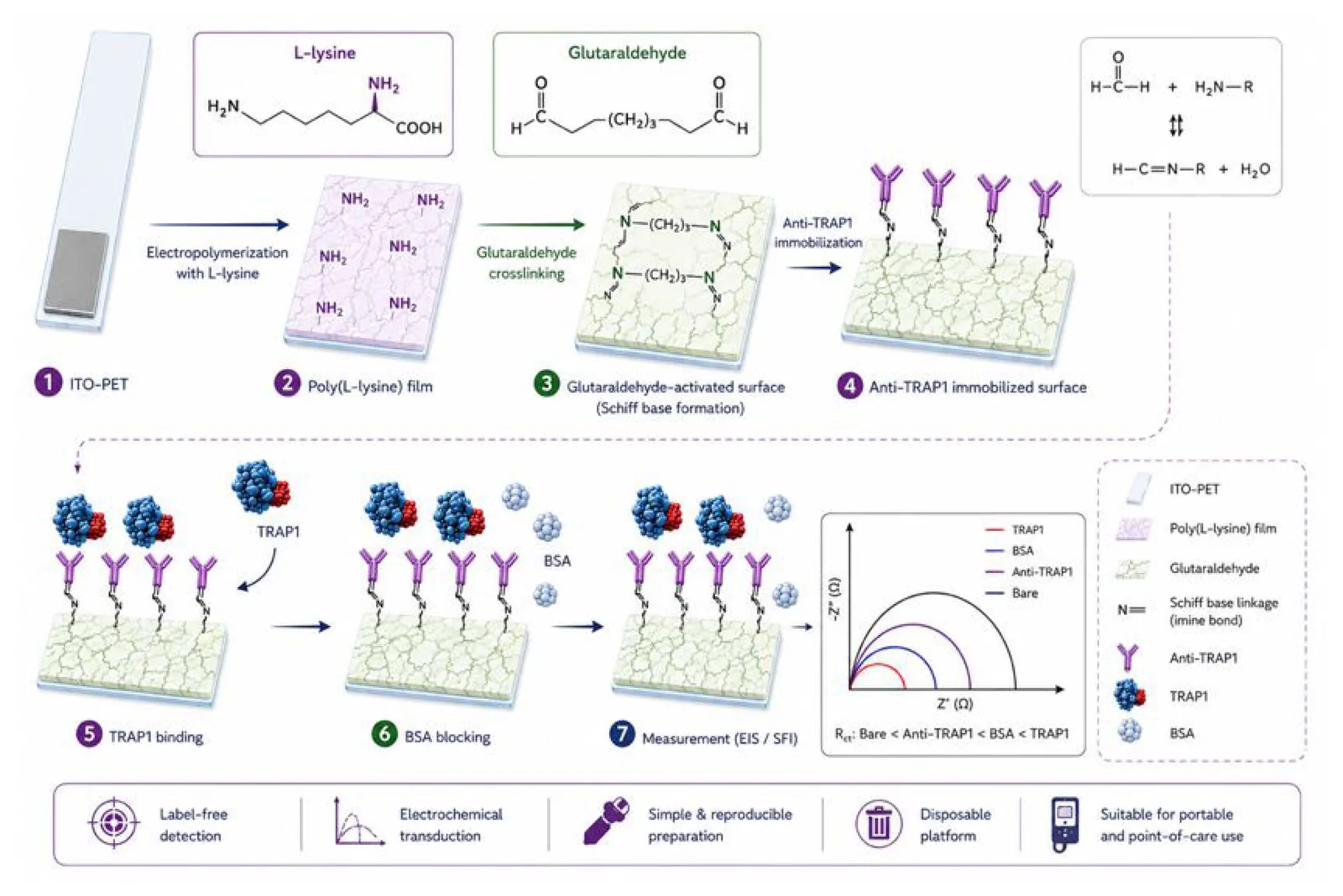
The immobilization strategies of the TRAP-1 biosensor.

**Table:** The analysis of TRAP-1 in commercial human serum samples

Serum	Determined TRAP-1 by biosensor (pg/mL)	Added TRAP-1 concentration (pg/mL)	Total found TRAP-1 by biosensor (pg/mL, n=3)	RSD% (n=3)	Recovery (%)

1	4.769	0.5	**5.49/5.28/5.21**	**1.01**	**101.01**
		2	**7.24/6.77/6.87**	**2.82**	**102.82**

2	0.493	0.5	**1.09/1.08/0.93**	**4.16**	**104.16**
		2	**2.32/2.56/2.58**	**0.23**	**99.77**

**3**	12.869	0.5	**13.44/13.53/13.30**	**0.40**	**100.4**
		2	**14.91/14.77/14.59**	**0.76**	**99.24**

4	0.218	0.5	**0.69/0.82/0.63**	**0.43**	**99.57**
		2	**2.31/2.46/2.00**	**4.81**	**104.81**

5	0.752	0.5	**1.15/1.26/1.28**	**1.68**	**98.32**
		2	**2.77/2.75/2.83**	**1.05**	**101.05**

## Footnotes

1 World Health Organization (WHO) (2024). Global Cancer Burden Growing, Amidst Mounting Need for Services [online]. Website https://www.who.int/news/item/01-02-2024-global-cancer-burden-growing--amidst-mounting-need-for-services [accessed 23 April 2026].
